# Arsenic and Cancer

**Published:** 1902-07-26

**Authors:** 


### ARSENIC AND CANCER.

Notwithstanding the attempts made by some of his surgical friends to throw ridicule upon his hypothesis, Mr. Jonathan Hutchinson sticks to the idea that the gradually-increasing dissemination of arsenic which results from the artificiality of modern life may have some causal relation to the gradually-increasing prevalence of cancer which is stated to have taken place during recent years in progressive countries. In this month's *Polyclinic*, of which he is the editor, an article appears under the heading "Arsenic and Cancer," which says: "The suggestion that arsenic has played a part, and possibly not an unimportant one, in the recent increase of cancer, finds suggestive illustrations on all sides. Dr. Solomon Smith writes to us to ask attention to the fact that soot contains arsenic, and that in this way its well-known influence in producing cancer may now be explained. Should this be accepted, a paragraph in an article on cancer in the new volume of the *Encyclopædia Britannica* will read almost like a prophecy. After discussing the influence of vocation upon the incidence of cancer, Dr. Shadwell concludes by saying that nothing is proved excepting the one fact that chimney-sweeps are far more liable to it than others. He adds: The soot is supposed to act as an irritant; but, if so, why are potters and coal-miners, who also work in irritating materials, so very low in the cancer scale? No doubt the case of the chimney-sweep contains one of the keys to the problem of cancer causation, but it has not been found yet. Now, possibly, in the suggestion as to arsenic, the key has been found." The suggestion at present made as regards arsenic is, it says, that whether taken medicinally or dietetically, or inhaled as dust, or applied externally to the skin, it has the effect, often without producing other symptoms of derangement, of predisposing the tissues to cancerous modes of growth. This, of course, falls very far short of what some of the daily papers which have commented upon Mr. Hutchinson's remarks have attributed to him.

